# Safety Profile of Niraparib as Maintenance Therapy for Ovarian Cancer: A Systematic Review and Meta-Analysis

**DOI:** 10.3390/curroncol29010029

**Published:** 2022-01-12

**Authors:** Antonia Pagkali, Ioannis Mamais, Adamantios Michalinos, Aris P. Agouridis

**Affiliations:** 1 School of Medicine, European University Cyprus, Nicosia 2404, Cyprus; onco.toniapagkali@gmail.com (A.P.); a.michalinos@euc.ac.cy (A.M.); 2 Department of Health Sciences School of Sciences, European University Cyprus, Nicosia 2404, Cyprus; i.mamais@euc.ac.cy

**Keywords:** niraparib, maintenance therapy, safety profile, meta-analysis, ovarian cancer, randomised controlled trials

## Abstract

Background: Patients with epithelial ovarian cancer (EOC), treated with niraparib maintenance, present with haematological and gastrointestinal toxicities. Limited data exist on niraparib safety assessment. Objective: To evaluate niraparib safety profile, as maintenance therapy, in women with platinum-sensitive EOC. Methods: PubMed and Cochrane searches were carried out up to April 2021 for randomised controlled trials (RCTs) evaluating niraparib versus placebo in EOC patients with a response to platinum-based chemotherapy. Regarding the meta-analysis, for dichotomous data, the pooled risk ratio (RR) was calculated. Results: A total of 1539 patients from three RCTs revealed that niraparib-treated patients are associated with a significantly higher risk of any grade of nausea (RR, 2.15; 95% CI, 1.86 to 2.48), fatigue (RR, 1.26; 95% CI, 1.05 to 1.52, *p* < 0.00001), anemia (RR, 6.86; 95% CI, 2.54 to 18.52, *p* = 0.0001), thrombocytopenia (RR, 7.02; 95% CI, 1.68 to 29.38, *p* < 0.00001), vomiting (RR, 2.51; 95% CI, 1.50 to 4.19, *p* = 0.0005), neutropenia (RR, 2.96; 95% CI, 1.13 to 7.73, *p* < 0.00001), headache (RR, 2.08; 95% CI, 1.57 to 2.74, *p* < 0.00001), constipation (RR, 2.10; 95% CI, 1.72 to 2.57, *p* < 0.00001) and insomnia (RR, 2.48; 95% CI, 1.52 to 2.89, *p* = 0.0003) when compared with placebo. For grade 3 or 4 adverse effects, significantly higher risk was only noted for fatigue (RR,6.25; 95% CI, 1.70 to 23.05, *p* = 0.006), anemia (RR, 16.23; 95% CI, 4.86 to 54.17, *p* < 0.00001), thrombocytopenia (RR, 35.12; 95% CI, 12.23 to 100.82, *p* < 0.00001) and neutropenia episodes (RR, 6.35; 95% CI, 2.08 to 19.39, *p* = 0.001) for those taking niraparib. Notably, incidents of adverse effects and discontinuation rates were substantially lower among patients treated with an individualised niraparib dose than those treated with the standard one. Efficacy was not reduced, and no treatment-related deaths occurred during the included trials. Conclusion: Niraparib is considered an effective and well-tolerated choice, with an improved safety profile, for the maintenance treatment of EOC patients.

## 1. Introduction

Ovarian cancer is a heterogenous group of neoplasms, which represents 1.2% of all cancers [1]. About 70% of patients with advanced epithelial ovarian cancer (EOC), in remission after cytoreductive surgery and platinum-based chemotherapy, will eventually relapse, regardless of their previous response to treatment [2].

The current established approach for treating relapses in complete platinum-sensitive or partially sensitive EOC patients is a second round of chemotherapy and, whenever necessary, repeated surgery [2]. However, these approaches present some toxicities [2]. The only approach that recently presented with great potential against tumor development and a statistically significant improvement in progression-free survival (PFS) was bevacizumab [3,4], a humanised antibody, which neutralises vascular endothelial growth factor, inhibiting blood vessels formation. Unfortunately, bevacizumab is also associated with several adverse events such as hypertension, thromboembolism and proteinuria [5].

This narrow range of therapeutic choices and the limited number of drugs available for maintenance therapy emphasised the need for strategies that prevent relapses, improve PFS and ameliorate the clinical benefit for EOC patients. In this landscape, the inhibitors of poly (ADP-ribose) polymerases (PARP inhibitors) have demonstrated a dramatic anti-tumor effect, for a number of tumors, and are currently emerging as a gold-standard therapeutic option [6]. Nowadays, PARP inhibitors are starting to be validated as maintenance therapy, after completion of chemotherapy treatment, by prolonging remission and by preventing disease progression [6].

Niraparib (Zejula^®^, GLAXOSMITHKLINE company, London, UK) was approved by the Food and Drug Administration in March of 2017 for patients with recurrent EOC, fallopian tube or primary peritoneal cancer that previously responded well to platinum-based chemotherapy [7]. Like other PARP inhibitors, niraparib induces cell cycle arrest in the G2/M phase of cancer cells, followed by apoptosis and mitosis dysregulation [8]. In vitro, the anti-tumor activity of niraparib was observed across all tumor cell lines, regardless of mutations [8]. The era of niraparib comes with many advantageous characteristics, targeting the inherent deficiencies of cancer cells while preserving healthy functioning cells [9]. Niraparib presents a manageable tolerability profile in patients with ovarian, fallopian tube or primary peritoneal cancer [9]. The most reported adverse effects include haematological and gastrointestinal toxicities [9]. Dosage reduction is the method of choice adopted, in order to minimise any decrease in quality of life (QoL) [9].

The aim of this study is to perform a systematic review and a meta-analysis of randomised controlled trials (RCTs), on the safety of the single-agent niraparib, as the maintenance therapy in patients with platinum-sensitive EOC compared with placebo.

## 2. Methods

### 2.1. Study Design

We performed a qualitative synthesis of published RCTs to evaluate the safety profile of niraparib, which lengthens PFS in patients with EOC, after the completion and response to platinum-based chemotherapy cycles. Additionally, a meta-analysis on niraparib’s any grade and grade 3 or 4 adverse effects of the included studies was conducted.

### 2.2. Searching

An extensive bibliographic search of PubMed and Cochrane database was conducted until 10 April 2021. We identified a timeframe between August 2013 and April 2021. Initial searches were carried out using the following search terms: (“niraparib”) AND (“random*” OR “controlled trial” OR “clinical trial” OR “randomised controlled trial” OR “placebo” OR “double-blind”) AND (“ovarian cancer”). Similar articles retrieved from the reference lists of studies, which investigated niraparib among ovarian cancer patients, were also used for further searches. For this search, only papers based on humans and written in the English language were considered. The protocol was registered with the international prospective register of systematic reviews (PROSPERO), in accordance with PRISMA-P guidelines (PROSPERO CRD42021286446).

### 2.3. Eligibility Criteria

Inclusion criteria for our systematic review and meta-analysis encompassed phase III RCTs comparing niraparib versus placebo as maintenance therapy in patients with high-grade EOC, either newly diagnosed or relapsed. We excluded noncomparative, non-randomised (e.g., case controls, cohorts or cross-sectional) and studies not written in English.

### 2.4. Data Extraction

**Figure 1:**
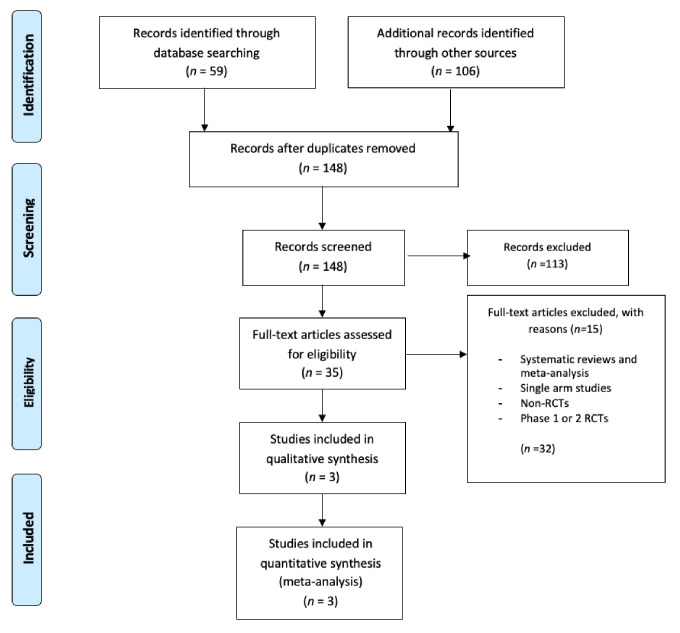
PRISMA chart.

Studies were independently assessed by two investigators (A.P., A.P.A.) and the study characteristics were extracted. Any discrepancy between the reviewers was resolved by consensus. We evaluated study characteristics (e.g., first author, publication year, study design and trial phase), patient characteristics (e.g., median age, number enrolled, cancer type, mutations, treatment schedule and dosage; frequency of administration) and clinical outcomes (median PFS and overall survival (OS)). For the review of our analysis, which was designed according to the guidelines of 2009, data extraction was performed with adherence to Preferred Reporting Items for Systematic reviews and Meta-Analysis (PRISMA model, Figure 1). Due to the study design, neither Institutional Review Board (IRB) approval, nor patients’ informed consents were required. The initial searches for relevant reports were carried out by using the basis of brief title/abstract review and by removing duplicates where possible. Next, articles were selected based on later examination of full-text articles to reach our final decision.

### 2.5. Assessment Risk of Bias

A systematic assessment of bias, in the included studies, was performed using the Cochrane ROB 2 tool [10]. The items used for the assessment of each study were as follows: adequacy of sequence generation, allocation concealment, blinding of subjects and personnel, blinding of outcome assessment, addressing of dropouts (incomplete outcome data), selective outcome reporting and other potential sources of bias. According to the recommendations of the Cochrane Handbook, a judgment of “yes” indicated low risk of bias, while “no” indicated high risk of bias. Labeling an item as “unclear” indicated an unclear or unknown risk of bias. Risk of bias assessment was performed independently by 3 reviewers (A.P., A.P.A., I.M.); disagreements were resolved by consensus. According to the ARMSTAR2 tool [11], in order to appraise this systematic review of randomised controlled clinical trials, our study was identified as a high-quality review paper.

### 2.6. Data Analysis

Where sufficient information was obtainable and the outcome measures were comparable, meta-analysis was performed, allowing a quantitative analysis of the studies. The pooled estimations regarding outcomes were expressed as dichotomous.

This was calculated using a random-effect model or a fixed-effect model. For dichotomous data, the pooled risk ratio (RR) was calculated. Subgroup analysis was performed according to AE. Statistical analysis was performed using the Review Manager (RevMan) Version 5.0 software (The Nordice Cochrane Center, The Cochrane Collaboration, Copenhagen, Denmark, 2008) and STATA Version 13. *p* < 0.05 was considered significant.

### 2.7. Heterogeneity Analysis

The existence of statistical heterogeneity between the included studies was assessed using the I^2^ test. The heterogeneity was considered low, moderate, or high if the I^2^ was 25, 50 or >75%, respectively. If the *p*-value was less than 0.10, the random effect model been adopted or vice versa. The between-trial heterogeneity was assessed using the Q test and the I^2^ statistic.

## 3. Results

Three RCTs of 1539 patients were randomised, with either niraparib or placebo maintenance, in our analysis [12,13,14]. Baseline characteristics are summarised in Table 1. The study is categorised as low risk of bias, implying confidence that results represent true treatment and adverse effects. Findings are likely to be reliable, since no major or minor sources of bias are likely to influence our results, supporting our conclusions by evidence (Supplementary Figure S1).

### 3.1. Study Selection

In Figure 1, PRISMA chart reveals how the selection of our studies was made. With the appropriate search terms, we identified 59 records on PubMed and 106 additional records on Cochrane, which were all reported as full papers. After detecting and removing duplicates, we had 148 articles of which we initially excluded 113 because of reviews and trial design. Subsequently, we examined in detail the remaining 35 trials. Among them, 32 trials were rejected because the selection criteria were not met. Lastly, three RCTs with 1551 patients were included in our final analysis. Of them, a total of 1539 patients were assigned to either niraparib or placebo. PRISMA checklist is included in the supplementary material (Supplementary Table S1).

### 3.2. Dosage Modification Analysis

In the NOVA trial [12], treatment related adverse events of any grade were seen in 97.5% of patients treated with niraparib and in 70.9% of those treated with placebo. More specifically, the incidence of grade 3 or 4 adverse events in the niraparib group was substantially higher, noting 74.1% of its population versus the 22.9% of the placebo cohort. Among niraparib-treated patients, the most reported any grade adverse events included nausea (73.6%), thrombocytopenia (61.3%), fatigue (59.4%) and anemia (50.1%) whereas the most reported grade 3 or 4 adverse events were thrombocytopenia (33.8%), anemia (25.3%), neutropenia (19.6%) and fatigue (8.2%). Initial dose was 300 mg for all patients. However, in case of unbearable toxicities and based on physicians’ clinical judgment, treatment interruption for a short period of time (up to 28 days) and/or dose reduction could have been implemented, but no more than two dosage reductions were permitted (200 and 100 mg/day). Dosage modification, due to treatment-related adverse effects, was noted in 66.5% for patients on niraparib, and treatment discontinuation in 14.7%. Assessment of patients’ quality of life, from both the controlled and placebo group, revealed that outcomes were the same. No treatment-related deaths occurred during the trial.

In the PRIMA study [13] treatment-related adverse events of any grade were seen in 96.3% of niraparib treated patients and in 68.9% of placebo treated ones. More specifically, the incidence of grade 3 or 4 adverse events in the control group was substantially higher, noting a 65.3%, versus the 6.6% of the population taking placebo. Among niraparib-treated patients, the most reported any-grade adverse events included thrombocytopenia with platelet count decreased (73.3%), anemia (63.4%) and nausea (57.4%), whereas the most-reported grade 3 or 4 adverse events were thrombocytopenia with platelet count decreased (41.7%), anemia (31%), and neutropenia (12.8%). Initial dose was 300 mg for all patients. However, after the trial’s first protocol amendment, patients were administered with either a fixed (300 mg/day) or an individualised dose of niraparib maintenance or placebo. Patients treated with the individualised dose were given either 300 or 200 mg/day of treatment based on their baseline body weight or platelet count number (300 mg/day for those with baseline body weight ≥ 77 kg and platelet count ≥ 150,000 μL, 200 mg/day for those with baseline body weight < 77 kg or platelet count < 150,000 μL). In total, dosage reduction was observed in 70.9% of the cases who were treated with niraparib, and treatment discontinuation in 12%. Notably, it was also observed that after the protocol’s amendment and the initiation of the individualised dosing, any grade and grade 3 or 4 adverse effects were considerably decreased compared with the group receiving the fixed starting dose. No related to treatment deaths were occurred during the trial.

In the NORA study [14], treatment related adverse events of any grade were seen in 99.4% of patients treated with niraparib versus 87.5% for those treated with placebo. Particularly, the incidence of grade 3 or 4 adverse events in the niraparib group occurred in 44.6% of the population, versus the 11.4% in the placebo cohort. Among niraparib-treated patients, the most-reported any-grade adverse events included white blood cell count decreased (59.3%), neutropenia (58.8%), platelet count decreased (54.8%), anemia (53.1%) and nausea (53.1%) whereas the most reported grade 3 or 4 adverse events were neutropenia (20.3%), anemia (14.7%) and platelet count decreased (11.3%). Starting dose was 300 mg/day for the first 16 enrolled patients treated with niraparib or placebo. However, shortly after protocol’s modification, the rest of the patients were assigned to an individualised starting dose based on their bodyweight and platelet count. Most of them, who had a bodyweight < 77 kg or a platelet count < 150,000 μL, were treated with 200 mg/day, while those with baseline body weight ≥ 77 kg and platelet count ≥ 150,000 μL received 300 mg/day of niraparib or matched placebo maintenance. Dosage modification, due to treatment related adverse effects, was detected in 59.9% for patients on niraparib and treatment discontinuation in only 4%. No patient’s quality of life assessment has been made. No related to treatment deaths were occurred by the time of trial’s primary cut-off date, although only one similar scenario was observed after the predefined date.

### 3.3. Meta-Analysis of Any Grade and Grade 3 or 4 Adverse Effects

**Figure 2:**
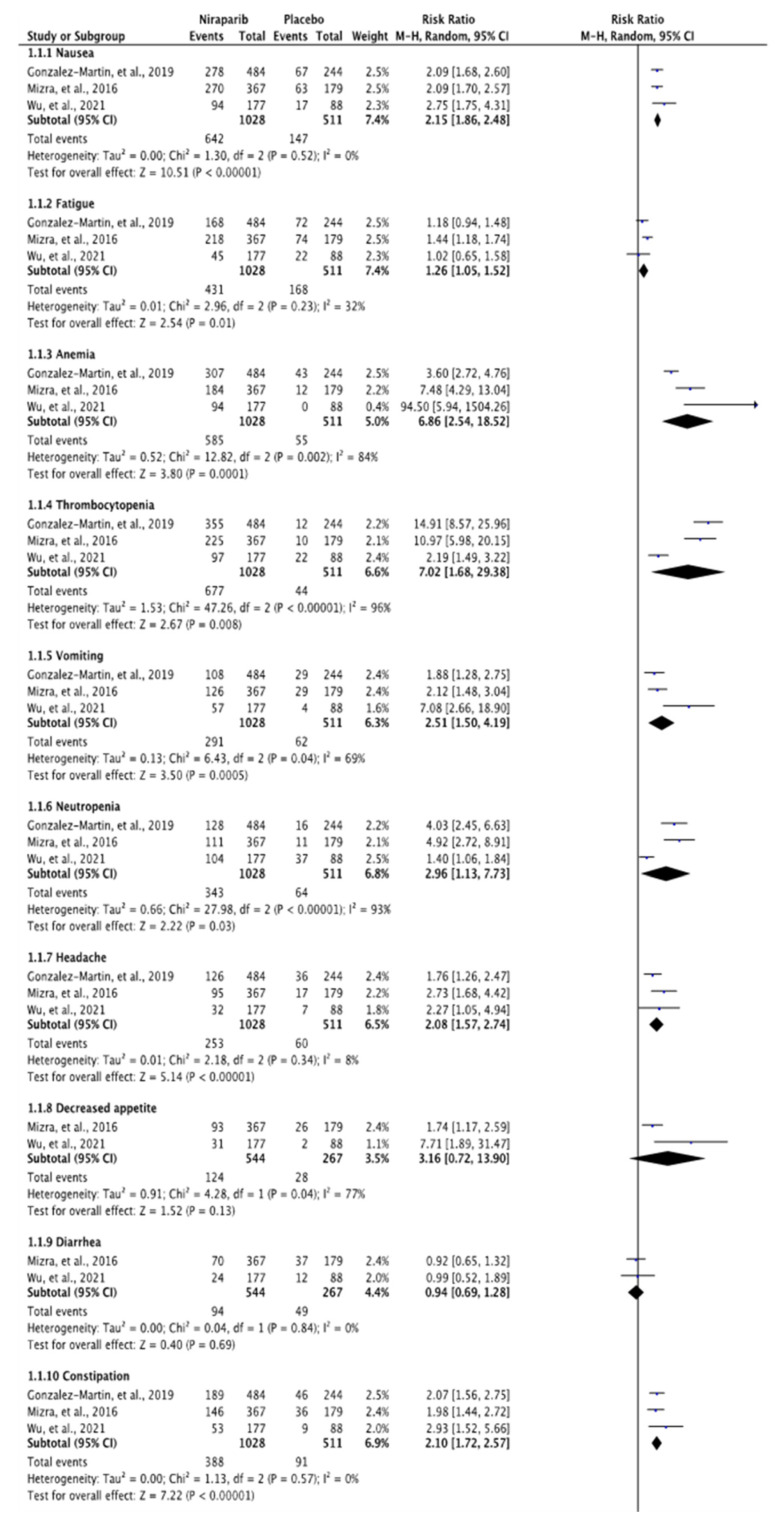

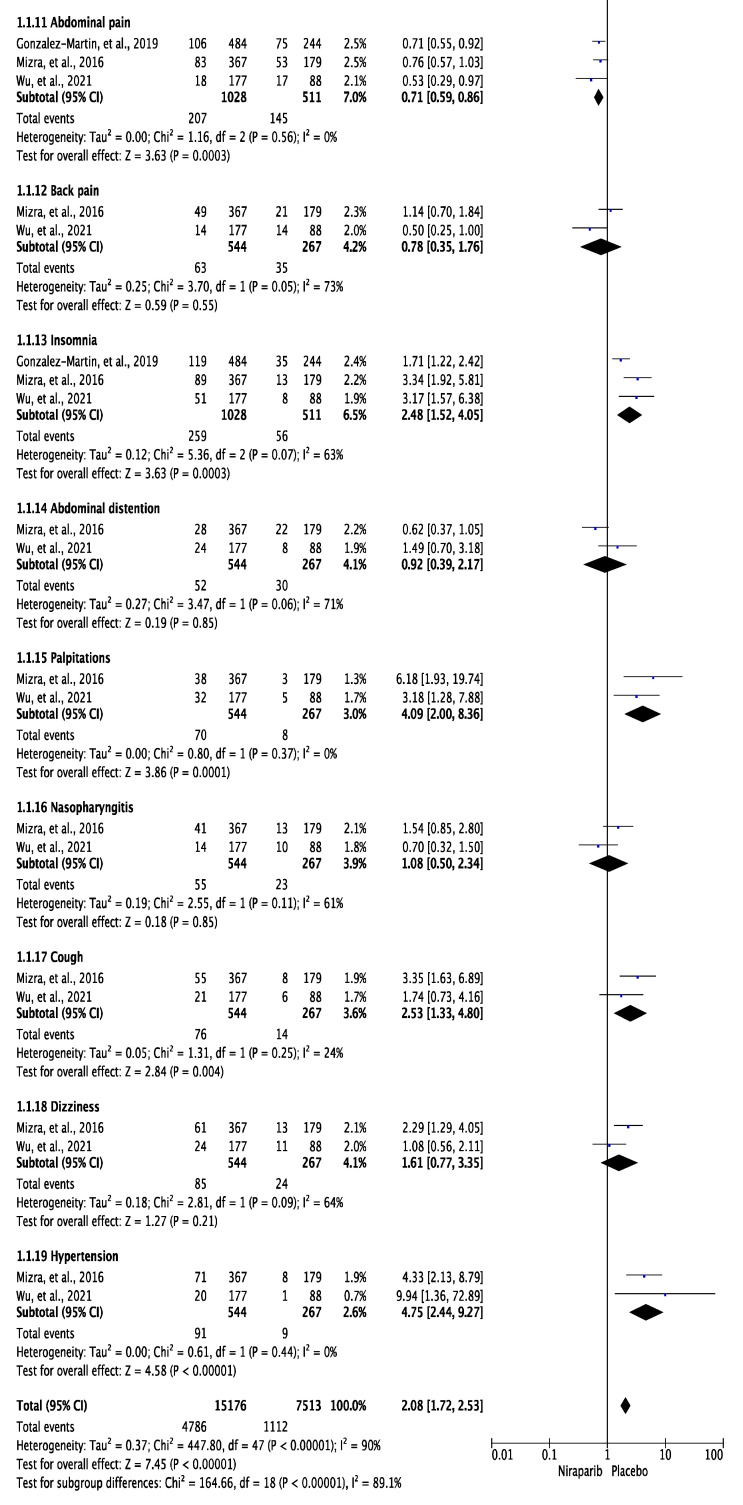
Forest plot of the meta-analysis of any grade adverse effects in NOVA, PRIMA and NORA studies.

**Figure 3:**
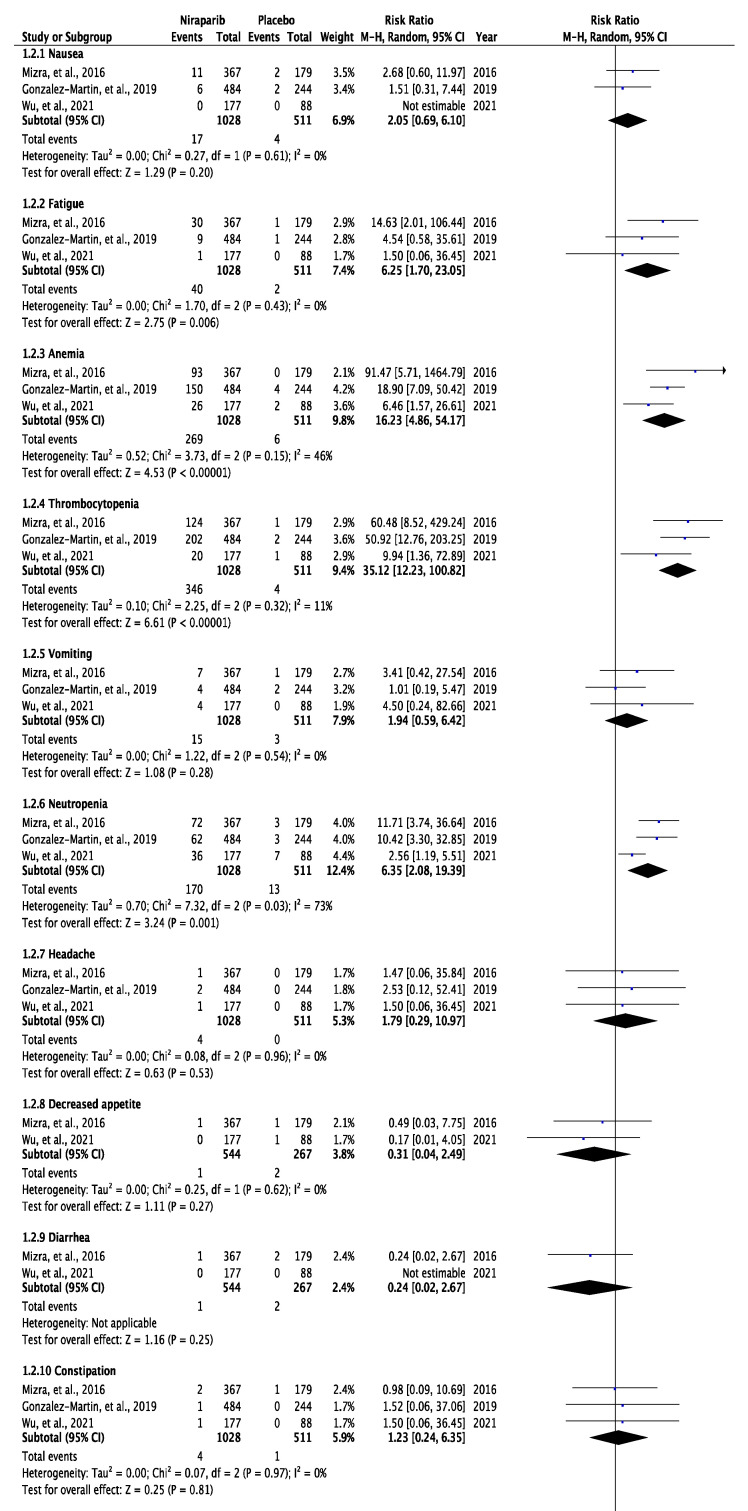

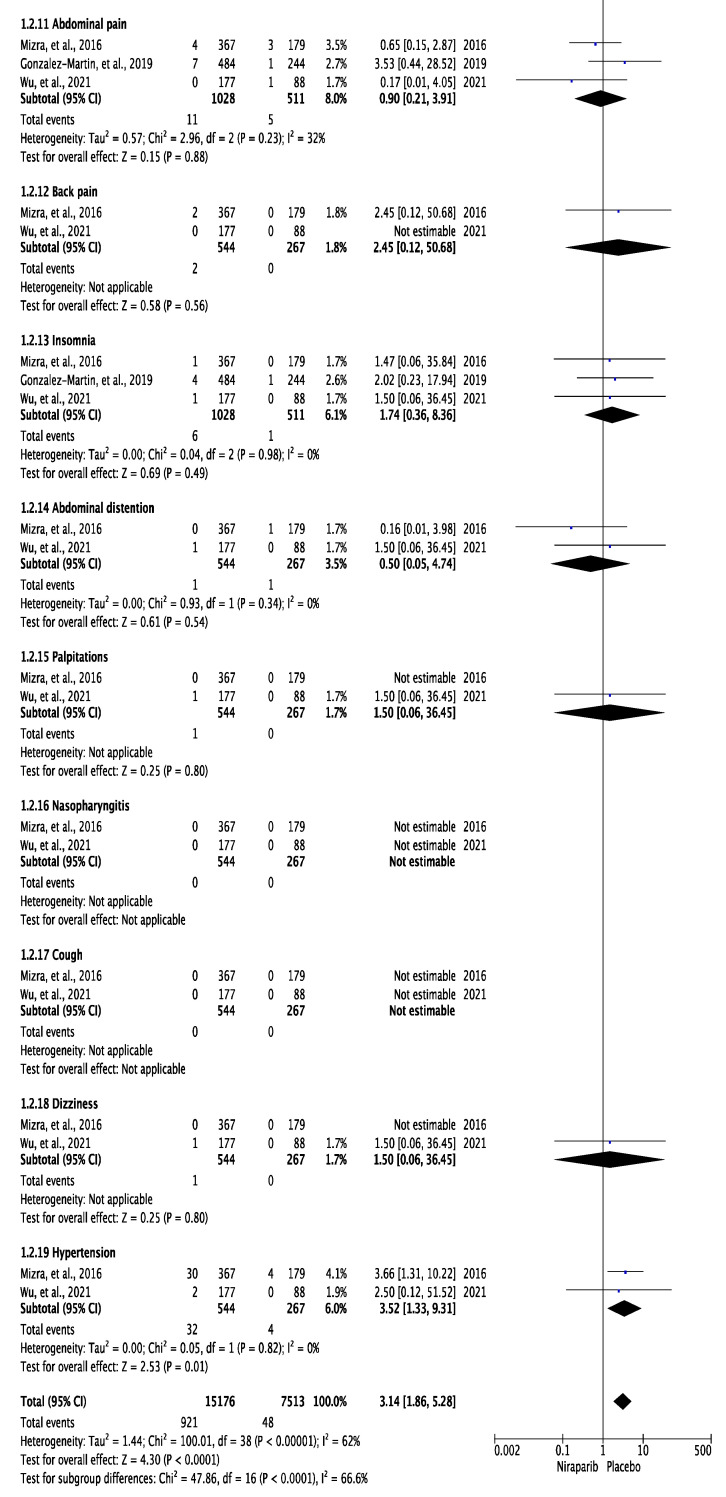
Forest plot of the meta-analysis of grade 3 or 4 adverse effects in NOVA, PRIMA and NORA studies.

Adverse effects were recorded in 3/3 studies [12,13,14] and the results of our meta-analysis are summarised in Figure 2 and Figure 3. In the total 1028 cases of niraparib, there were 642 incidences of nausea of any grade, while from the 511 cases of placebo there were 147 cases mentioned (RR, 2.15; 95% CI, 1.86 to 2.48, *p* < 0.00001). As for the rest of the gastrointestinal disturbances, 291 from 1028 patients presented with vomiting episodes, while in the placebo group only 62 incidents out of 511 patients were recorded (RR, 2.51; 95% CI, 1.50 to 4.19, *p* = 0.0005). Patients treated with niraparib encountering constipation issues were counted to 388, while those taking placebo were 91 (RR, 2.10; 95% CI, 1.72 to 2.57, *p* < 0.00001). Abdominal pain of any grade was mentioned in 207 patients taking niraparib and in the placebo group, 145 did experience abdominal pain of any grade (RR, 0.71; 95% CI, 0.59 to 0.86, *p* = 0.0003). As for the haematological adverse effects, 585 patients treated with niraparib versus 55 placebo patients presented with anaemia (RR, 6.86; 95% CI, 2.54 to 18.52, *p* = 0.0001). Thrombocytopenia of any grade was recorded in 677 niraparib cases, while in the placebo group there were only 44 cases (RR, 7.02; 95% CI, 1.68 to 29.38, *p* = 0.008). Similarly, neutropenia was recorded in 343 niraparib-treated patients and in only 64 cases in the placebo group (RR, 2.96; 95% CI, 1.13 to 7.73, *p* < 0.00001). The rest of the symptoms which were recorded from patients in all three studies included fatigue (RR, 1.26; 95% CI, 1.05 to 1.52, *p* = 0.01), headache (RR, 2.08; 95% CI, 1.57 to 2.74, *p* < 0.00001) and insomnia (RR, 2.48; 95% CI, 1.52 to 4.05, *p* = 0.0003). Incidents of any grade such as decreased appetite, diarrhoea, back pain, abdominal distention, palpitations, nasopharyngitis, cough, dizziness and hypertension were only recorded among the NOVA [11] and the NORA [13] trials (Figure 2, Supplementary Table S2).

Regarding the grade 3 or 4 adverse effects, a higher risk of episodes was observed in the group of patients treated with niraparib. We counted 17 patients out of a total 1028 treated with niraparib with grade 3 or 4 nausea, while in the placebo group, only 4 out of 511 were experiencing the same (RR, 2.05; 95% CI, 0.69 to 6.10, *p* = 0.20). Grade 3 or 4 vomiting was seen in 15 out of 1028 patients taking niraparib and in 3 out of 423 taking placebo (RR, 1.94; 95% CI, 0.59 to 6.42, *p* = 0.28). Grade 3 or 4 constipation was recorded in 4 niraparib-treated patients, while in only one case in the placebo cohort (RR, 1.23; 95% CI, 0.24 to 6.35, *p* = 0.81). Moreover, 11 patients taking niraparib and 5 taking placebo did experience grade 3 or 4 abdominal pain episodes (RR, 0.90; 95% CI, 0.21 to 3.91, *p* = 0.88). As for the grade 3 or 4 haematological adverse effects, anaemia was noted in 269 patients taking niraparib, while the placebo group counted only six patients (RR, 16.23; 95% CI, 4.86 to 54.17, *p* < 0.00001). Thrombocytopenia-recorded episodes were 346 in the niraparib group and 4 in the placebo cohort (RR, 35.12; 95% CI, 12.23 to 100.82, *p* < 0.00001). Neutropenia grade 3 or 4 was seen in 170 patients in the niraparib group, while 13 patients presented with severe neutropenia among the placebo group (RR, 6.35; 95% CI, 2.08 to 19.39, *p* = 0.001). The rest of grade 3 or 4 adverse effects that were noted in all three studies included fatigue (RR,6.25; 95% CI, 1.70 to 23.05, *p* = 0.006), headache (RR,1.79; 95% CI, 0.29 to 10.97, *p* = 0.53), constipation (RR,1.23; 95% CI, 0.24 to 6.35, *p* = 0.81), abdominal pain (RR,0.90; 95% CI, 0.21 to 3.91, *p* = 0.88) and insomnia (RR,1.74; 95% CI, 0.36 to 8.36, *p* = 0.49). Incidents of grade 3 or 4 such as decreased appetite, diarrhoea, back pain, abdominal distention, palpitations, nasopharyngitis, cough, dizziness and hypertension were only recorded among the NOVA [12] and the NORA [14] trials (Figure 3, Supplementary Table S3).

## 4. Discussion

This study includes the only three currently available phase III RCTs comparing niraparib versus placebo in patients with platinum sensitive high-risk EOC. The study’s aim is to strengthen the evidence in niraparib’s favor, as both an effective and a relatively safe agent for the maintenance therapy. The meta-analysis was conducted in order to investigate the relative risks (RR) of all-grade and grade 3 or 4 adverse events presented in the included clinical trials. Previous studies have already proved that PARP inhibitors, such as olaparib [15,16,17] and rucaparib [18], are valuable treatment options for patients with BRCA-mutated ovarian cancer. Our study’s results came in accordance with the most reported adverse events of previous PARP inhibitors studies, discussed in detail later.

Dosage modifications during the NOVA [12], PRIMA [13] and NORA [14] studies, due to treatment related adverse effects, were noted in 66.5, 70.9 and 59.9% of niraparib treated cases, respectively. In the retrospective analysis [19], for the safety and dosage modification of patients in the NOVA [12] trial, it was validated that baseline bodyweight < 77 kg or platelet number < 150,000 μL were the decisive factors for administering patients with a reduced dose of maintenance, without jeopardising treatment efficacy. Based on this subanalysis [19], it was revealed that thrombocytopenia was the most prevailing adverse effect that led patients to dosage reduction, and due to that, the most prescribed dosage was 200 mg/day. Furthermore, most high-grade adverse effects occurred within the first 3 months from trial’s initiation; these adverse effects included grade 3 or 4 thrombocytopenia (33%), neutropenia (18%) and anemia (15%). Notably, grade 3 or 4 thrombocytopenic events reached a 0.7% and neutropenia reached 1.6% for the patients shifting to 200 mg/day of niraparib maintenance. In addition to that, results also showed that the any-grade thrombocytopenia rate was 59.9% for those taking niraparib 300 mg/day, compared with 35.4% for those treated with 200 mg/day. In a likewise manner, fatigue of 300 mg/day and 200 mg/day was counted to 34.1 and 26% respectively, while nausea incidents were esteemed to 67.8 and 26.8%, respectively. Overall, when patients achieved an either 200 or 100 mg/day dosage of treatment maintenance, PFS was still consistent with that of patients treated with 300 mg/day standard dose. For all those reasons mentioned above, PRIMA trial [13] protocol was later reconsidered, and physicians administered patients with either a fixed (300 mg/day) or an individualised dose (300 or 200 mg/day) of niraparib maintenance or placebo, based on patients’ bodyweight and platelet count. In a similar manner, phase III NORA study [14] has adopted from the beginning the amended protocol of individualised starting dosage based on the predefined elements. In this study, not only we observed a decline in the occurrence of grade 3 or 4 adverse effects for those treated with niraparib, where the individualised dose was initiated in 94% of the patients enrolled, but discontinuation rate was extremely low in comparison with the two other trials.

Having all things considered, in all three trials, niraparib maintenance was well tolerated, and no reduction in efficacy was seen, despite patients’ treatment with the reduced dosage of niraparib or placebo. Since during the NOVA [12] and PRIMA [13] trials, a categorisation has been made based on patients’ mutation status, it is complicated to compare median PFS with that of NORA study [14], where such a patients’ classification was not seen. However, it is remarkable to mention that BRCA-mutated patients in NORA [14] trial had a reduced risk of disease progression and death ((PFS had not yet reached versus 5.5 months; HR = 0.22; 95% CI, 0.12–0.39)) compared with the same population sample of the NOVA trial [11] ((21.0 vs. 5.5 months; HR = 0.27; 95% CI, 0.17–0.41)). In addition to that, non-germline BRCA-mutated patients also had a reduced risk of disease progression and death ((PFS 11.1 months versus 3.9 months; HR = 0.40; 95% CI, 0.26–0.61)) compared with the same population sample of the NOVA trial [11] ((PFS 9.3 months versus 3.9 months; HR = 0.45; 95% CI, 0.34 to 0.61; *p* < 0.001)).

A subanalysis [20] of the NOVA trial [12] aimed to evaluate more precisely the beneficial effect of niraparib in patients aged ≥70 years with EOC, since older patients might in generally present with an increased risk of cumulative toxicities. This subanalysis established that older patients, who received niraparib as maintenance, not only presented with an equally sufficient PFS as those aged <70 years old, but the frequency of adverse effects was proportional to the effects noted in the younger population. A similar study investigated the overall tolerability and toxicity of olaparib towards older patients, in comparison with the younger population [21]. In this study, olaparib’s results were concordant with the ones of niraparib; supplementary confirming the safety of PARP inhibitors among older patients. Furthermore, the use of niraparib also has the potential to increase the chemotherapy-free interval. This is proved extremely advantageous, since elderly patients are often unable to receive successive lines of chemotherapy, following progression, due to comorbidities [20]. As for the subanalysis of the NOVA trial [22], focusing on QoL, both niraparib and placebo cohorts exhibited similar outcomes. This subanalysis strongly suggested that maintenance treatment with niraparib did not alter patients’ QoL, since the PARP inhibitor did not adversely affect patient-reported outcomes, based on the answers given in the Functional assessment of cancer therapy Ovarian Symptoms Index (FOSI) and the European QoL five-dimension five-level questionnaire (EQ-5D-5L) questionnaires [22].

The era of PARP inhibitors surely comes with several adverse effects that need special attention. The use of niraparib is most associated with myelosuppressive and gastrointestinal side effect [23]. In our meta-analysis results, focusing on any-grade adverse effects, haematological toxicities including thrombocytopenia, anemia, neutropenia, and gastrointestinal toxicities including nausea, vomiting and constipation were most associated with niraparib maintenance and results were statistically significant. Furthermore, incidents such as fatigue, headache, and insomnia were presented in all our three studies and were most presented among the niraparib cohort with statistical significance. On the other hand, any-grade adverse effects such as diarrhea, abdominal pain, back pain, and abdominal distention presented with a higher risk among patients treated with placebo, nonetheless, results were not statistically significant. As for palpitations, cough and hypertension, a statistically significant higher risk was observed in the niraparib group, but incidents were only noted among two out of our three trials. What needs to be addressed is the fact that numerous any-grade adverse effects of our analysis presented with high heterogeneity, such as anemia (I^2^ 84%, *p* = 0.002), thrombocytopenia (I^2^ 96%, *p* = 0.00001), neutropenia (I^2^ 93%, *p* < 0.00001), decreased appetite (I^2^ 77%, *p* = 0.04), and back pain (I^2^ 73%, *p* = 0.05). Due to this heterogeneity, we opted to use the random effect model, which calculates confidence intervals more conservatively, rather than the fixed effect model does. This high heterogeneity is attributed mainly to the clinical differences between Caucasian and Asian population and earlier termination of the NORA [14] trial.

Considering grade 3 or 4 adverse effects, our meta-analysis results proved that niraparib maintenance therapy is associated with a statistically significant increased risk of fatigue, anemia, thrombocytopenia, and neutropenia. Grade 3 or 4 gastrointestinal adverse effects, including nausea, vomiting and constipation, were most reported among patients treated with niraparib; however, none of them presented a statistically significance. Notably, although grade 3 or 4 incidents of adverse effects were numerically less common, patients treated with niraparib had approximately 35 times more risk of presenting with grade 3 or 4 thrombocytopenia and 13 times more risk of grade 3 or 4 anaemia.

The meta-analysis of Guo et al. [24] evaluated olaparib monotherapy in advanced cancers and demonstrated an increase in the risk of severe anaemia compared with placebo (RR, 2.21; 95% CI, 1.53 to 3.49, *p* < 0.001). A most recent meta-analysis by Ruiz-Schutz et al. [25] revealed that treatment with olaparib, compared with other interventions, was associated with an increased risk of developing fatigue and anaemia, in line with our findings. Another recent meta-analysis of Zhou et al. [26] investigated the overall incidence and risk ratios of severe haematologic toxicities among patients treated with PARP inhibitors. This study demonstrated those patients had an increased risk of severe neutropenia, something that again comes in accordance with our 343 neutropenic cases, among which 170 were grade 3 or 4 for those taking niraparib. Likewise, regarding gastrointestinal toxicities, a meta-analysis by Liu et al. [27] has demonstrated that PARP inhibitor treatment is correlated with a significant increase in the risk of high-grade nausea and vomiting, with nausea being the most prevalent toxicity associated with olaparib administration [8]. However, these results do not agree with our meta-analysis results, since high-grade nausea and vomiting episodes were not statistically significant.

NOVA [12], PRIMA [13] and NORA [14] trials are undoubtedly three reliable clinical trials. However, there are some controversies that need to be discussed. The first inquiry arises from the fact that during the NORA [14] trial, there were no data considering patients’ homologous recombination status. The NORA [14] study presented with ameliorated drug tolerability, while preserving PFS, yet the number of patients with positive homologous recombination deficient status it is unknown. In addition to this, all the included studies were clinically heterogenous, different population groups of patients (patients with newly diagnosed or recurrent EOC), with a wide variation of mutations (plus those mutations who were not detected by the myChoice testing during the NOVA [12] and PRIMA [13] trials) and with discrepancies among the stage of their disease and previous treatments. All these attainable explanations might clarify some of the observed between-trial differences. Considering the NOVA subanalysis [20], for niraparib’s efficacy towards elderly patients, only 61 out of 553 total patients enrolled in the study who received niraparib were older than 70 years of age (*n* = 14 germline BRCA mutated and *n* = 47 non-germline BRCA mutated). This discrepancy may be due to the clinician bias against assigning elderly patients in clinical trials. In this subanalysis, 2/3 of the total treatment discontinuations among elderly patients happened due to grade 1 or grade 2 adverse effects. This explains why physicians and researchers sometimes avoid conducting studies including a great number of elderly patients. Despite of all these, we strongly believe in the reliability of our study and the certainty around its results, since all RCTs included a large population size.

Apart from the included trials, there are also some other significant interim studies, whose findings further confirm the conclusions of our review. A recent retrospective observational study [28], with a total of 153 patients diagnosed with recurrent ovarian cancer, had as an outcome of interest the rate of the three most-reported adverse effects (nausea, thrombocytopenia and fatigue) within the first 3 months of niraparib’s 200 mg/day initiation, compared with the patients of the NOVA trial [12] treated with 300 mg/day. More specifically, only 37% of them experienced at least one of the three adverse effects in a time of 3 months, out of whom 12% presented with a grade 3 or 4 adverse effect. It became evident that the prevalence of the most-reported adverse effects for patients treated with niraparib maintenance 200 mg/day was 2.5 to 4.5 times lower compared with patients treated with 300 mg/day in the NOVA trial [12]. Furthermore, the QUADRA study [29] revealed the statistically significant results of niraparib maintenance regarding PFS and its safety profile in women with high-grade EOC. It was excluded from our study, since it is a single-arm phase II clinical study, with a different objective of investigation. However, it is of great relevance to report that, the QUADRA study [29] findings support the continuum of niraparib monotherapy maintenance treatment, and no potential signals of serious adverse events were found. QUADRA [29] is one of the broadest clinical trials, which evaluated accurately the activity of the single-agent niraparib in the late-line treatment setting in a specific target population, legitimately comparable with the real-world patient population. In the post hoc safety and efficacy analysis [28] of the QUADRA [29] study, patients with low baseline bodyweight and platelet count had considerably higher rates of grade 3 or 4 thrombocytopenia (30%), neutropenia (12%) and anemia (9%) versus patients with ≥77 kg and ≥150,000 μL platelet count (14, 5, and 3%, respectively). The response rates to niraparib, as well as clinical benefit and disease-control rate, were comparable between patients treated with 300 and 200 mg/day, demonstrating that niraparib 200 mg/day presents with a reduced risk of toxicity yet a consistent PFS with those not dose-reduced.

In current practice, the use of PARP inhibitors, in the upfront setting, is not yet so commonly applicable. However, with more data available, there is an urge to question how PARP inhibitors, such as niraparib, will enter the clinical setting at any given time, in what dosage, how often and if so, in combination with which other currently available options. There have been multiple trials that support the combination of PARP inhibitors with novel agents, presenting a high response rate, such as phase I and II trials of niraparib in combination with bevacizumab [30,31] and single-arm phase I and II trial of niraparib with pembrolizumab [32].

## 5. Conclusions

After taking the above into consideration, niraparib represents a meaningful treatment option with a manageable tolerability profile for platinum-sensitive women with EOC. Niraparib extends patients PFS and we can nowadays consider it as an alternative option to maintenance therapy. Our findings are in line with the most frequently recorded adverse effects of previous PARP inhibitors studies. Although extended administration of niraparib may increase the risk of haematological and nonhaematological adverse events, our results suggest that it is a relatively safe agent; patients’ QoL was not compromised, and treatment-related adverse events were not correlated with any long-term morbidity or mortality. Our meta-analysis results proved that high-grade adverse effects of fatigue, anemia, thrombocytopenia, and neutropenia were the only incidents noted with a statistically significant higher risk among patients treated with niraparib versus placebo, yet these were considerably kept under control with the appropriate individualised dosage modifications, while preserving PFS. Adverse effects should be consistently managed so that patients can well tolerate the toxicities, receiving the appropriate dose for the most suitable amount of time. With the continuation of clinical trials and the initiation of the reduced niraparib-dosing regimen, niraparib will move forward into the forefront, as an effective and safe agent of EOC maintenance therapy.

## Figures and Tables

**Table 1:** Baseline Characteristics.

	NOVA Study Mirza et al. 2016 [12]				PRIMA Study González-Martín et al. 2019 [13]				NORA Study Wu et al. 2020 [14]	
	gBRCAm (+) 203 Total *n*. of patients		gBRCAm (−) 350 Total *n*. of patients		HRD (+) 373 Total *n*. of patients		Non-HRD(+) 249 Total *n*. of patients		177 Total *n*. of patients	88 Total *n*. of patients
	Niraparib	Placebo	Niraparib	Placebo	Niraparib	Placebo	Niraparib	Placebo	Niraparib	Placebo
Number of patients	138	65	234	116	247	126	240	120	177	88
Number of patients who discontinued treatment	91	61	188	104	126	83	184	93	101	77
Number of patients receiving ongoing treatment at data cut off	47	4	46	12	121	42	56	27	76	11
Median age, years	57	58	63	61	58	58	66	66	53	55
Primary location tumor										
Ovary	122	53	192	96	201	105	187	96	174	86
Other	16	12	42	19	46	21	53	24	3	2
Response to platinum-based chemotherapy										
Complete	71	33	117	60	185	93	152	79	121	60
Partial	67	32	117	56	62	33	88	41	56	28
Number to previous platinum-based regiments										
1	1	0	0	0	NR	NR	NR	NR	NR	NR
2	70	30	155	77	NR	NR	NR	NR	NR	NR
≥3	67	35	79	38	NR	NR	NR	NR	NR	NR

*n*: number, NR: none reported, gBRCAm (+): germline BRCA mutated positive, gBRCAm (-): germline BRCA mutated negative, HRD (+): homologous recombination deficient positive, Non-HRD (+): non- homologous recombination deficient positive.

## Supplementary Materials

The following are available online at https://www.mdpi.com/article/10.3390/curroncol29010029/s1, Figure S1: Risk of Bias (ROB2) summary; review authors’ judgments about risk of bias item for each included study in the review & Risk of Bias (ROB2) of included studies in percentage (intention-to-treat), Table S1: PRISMA Checklist 2009, Table S2: Any Grade Side Effects of NOVA, PRIMA and NORA studies, Table S3: Grade 3 or 4 Side Effects of NOVA, PRIMA and NORA studies.
