# Supplementary material for: Safety Profile of Niraparib as Maintenance Therapy for Ovarian Cancer: A Systematic Review and Meta-Analysis
Source: Curr Oncol. 2022 Jan 12;29(1):321–36. doi: 10.3390/curroncol29010029 (PMC8774559; doi:10.3390/curroncol29010029)
Supplement: Supplementary file 1 [file curroncol-29-00029-s001.zip › curroncol-1441071-supplementary.pdf]

Supplementary Materials

Studies with intention-to-treat	Unique ID	Study ID	Experimental	Comparator		Weight	Randomization process	Deviations from intended interve	Missing outcome data	Measurement of the outcome	Selection of the reported result	Overall
	1	ENGOT-OV16/NOVA	Niraparib	Placebo	PFS	1	+	+	+	+	+	+
	2	PRIMA/ENGOT-OV26	Niraparib	Placebo	PFS	1	+	+	+	+	+	+
	3	NORA	Niraparib	Placebo	PFS	1	+	+	+	+	+	+

\+ Low risk
? Some concerns
\- High risk

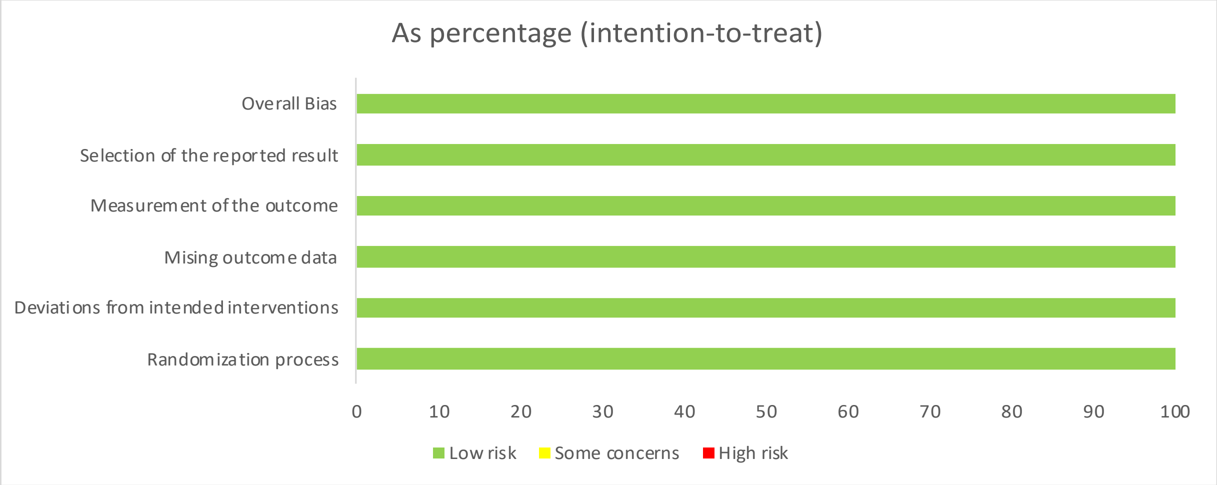

Figure S1. Risk of Bias (ROB2) summary; review authors' judgments about risk of bias item for each included study in the review & Risk of Bias (ROB2) of included studies in percentage (intention-to-treat).

Table S1. PRISMA Checklist 2009.

Section/Topic	#	Checklist Item	Reported on Page #
**TITLE**			
Title	1	Identify the report as a systematic review, meta-analysis, or both.	TITLE
**ABSTRACT**			
Structured summary	2	Provide a structured summary including, as applicable: background; objectives; data sources; study eligibility criteria, participants, and interventions; study appraisal and synthesis methods; results; limitations; conclusions and implications of key findings; systematic review registration number.	ABSTRACT
**INTRODUCTION**			
Rationale	3	Describe the rationale for the review in the context of what is already known.	INTRODUCTION
Objectives	4	Provide an explicit statement of questions being addressed with reference to participants, interventions, comparisons, outcomes, and study design (PICOS).	INTRODUCTION
**METHODS**			
Protocol and registration	5	Indicate if a review protocol exists, if and where it can be accessed (e.g., Web address), and, if available, provide registration information including registration number.	2.1 Study Design
Eligibility criteria	6	Specify study characteristics (e.g., PICOS, length of follow-up) and report characteristics (e.g., years considered, language, publication status) used as criteria for eligibility, giving rationale.	2.3 Inclusion and Exclusion Criteria
Information sources	7	Describe all information sources (e.g., databases with dates of coverage, contact with study authors to identify additional studies) in the search and date last searched.	2.2 Searching
Search	8	Present full electronic search strategy for at least one database, including any limits used, such that it could be repeated.	2.2 Searching
Study selection	9	State the process for selecting studies (i.e., screening, eligibility, included in systematic review, and, if applicable, included in the meta-analysis).	2.5 Study selection,
Data collection process	10	Describe method of data extraction from reports (e.g., piloted forms, independently, in duplicate) and any processes for obtaining and confirming data from investigators.	2.4 Data Extraction
Data items	11	List and define all variables for which data were sought (e.g., PICOS, funding sources) and any assumptions and simplifications made.	2.4 Data Extraction
Risk of bias in individual studies	12	Describe methods used for assessing risk of bias of individual studies (including specification of whether this was done at the study or outcome level), and how this information is to be used in any data synthesis.	2.6 Assessment Risk of Bias
Summary measures	13	State the principal summary measures (e.g., risk ratio, difference in means).	2.7 Data analysis,
Synthesis of results	14	Describe the methods of handling data and combining results of studies, if done, including measures of consistency (e.g., I^2) for each meta-analysis.	2.8 Heterogeneity analysis
Risk of bias across studies	15	Specify any assessment of risk of bias that may affect the cumulative evidence (e.g., publication bias, selective reporting within studies).	2.6 Assessment Risk of Bias
Additional analyses	16	Describe methods of additional analyses (e.g., sensitivity or subgroup analyses, meta-regression), if done, indicating which were pre-specified.	2.7 Data analysis & 2.8 Heterogeneity analysis
**RESULTS**			

Study selection	17	Give numbers of studies screened, assessed for eligibility, and included in the review, with reasons for exclusions at each stage, ideally with a flow diagram.	Figure 1
Study characteristics	18	For each study, present characteristics for which data were extracted (e.g., study size, PICOS, follow-up period) and provide the citations.	Table 1
Risk of bias within studies	19	Present data on risk of bias of each study and, if available, any outcome level assessment (see item 12).	Figure 2
Results of individual studies	20	For all outcomes considered (benefits or harms), present, for each study: (a) simple summary data for each intervention group (b) effect estimates and confidence intervals, ideally with a forest plot.	3.2 Meta- Analysis of Any Grade and Grade ¾ Adverse Effects, Tables 3 & 4, Figures 3 & 4
Synthesis of results	21	Present results of each meta-analysis done, including confidence intervals and measures of consistency.	3.2 Meta- Analysis of Any Grade and Grade ¾ Adverse Effects, Figures 3 & 4
Risk of bias across studies	22	Present results of any assessment of risk of bias across studies (see Item 15).	Figure 2
Additional analysis	23	Give results of additional analyses, if done (e.g., sensitivity or subgroup analyses, meta-regression [see Item 16]).	3.1 PFS analysis
**DISCUSSION**			
Summary of evidence	24	Summarize the main findings including the strength of evidence for each main outcome; consider their relevance to key groups (e.g., healthcare providers, users, and policy makers).	DISCUSSION
Limitations	25	Discuss limitations at study and outcome level (e.g., risk of bias), and at review-level (e.g., incomplete retrieval of identified research, reporting bias).	DISCUSSION
Conclusions	26	Provide a general interpretation of the results in the context of other evidence, and implications for future research.	CONCLUSION
**FUNDING**			
Funding	27	Describe sources of funding for the systematic review and other support (e.g., supply of data); role of funders for the systematic review.	Conflict of interest statement

Table S2. Any Grade Side Effects of NOVA, PRIMA and NORA studies.

	**NOVA STUDY 2016**		**PRIMA STUDY 2019**		**NORA STUDY 2021**	
	Niraparib (n=367)	Placebo (n=179)	Niraparib (n=484)	Placebo (n=244)	Niraparib (n=177)	Placebo (n=88)
NAUSEA ANY GRADE	270	63	278	67	94	17
†FATIGUE ANY GRADE	218	74	168	72	45	22
ANEMIA ANY GRADE	184	12	307	43	94	25
*THROMBOCYTOPENIA ANY GRADE	225	10	355	12	97	22
VOMITING ANY GRADE	126	29	108	29	57	4

NEUTROPENIA ANY GRADE	111	11	128	16	104	37
HEADACHE ANY GRADE	95	17	126	36	32	7
DECREASED APPETITE ANY GRADE	93	26	NR	NR	31	2
DIARRHEA ANY GRADE	70	37	NR	NR	24	12
CONSTIPATION ANY GRADE	146	36	189	46	53	9
ABDOMINAL PAIN ANY GRADE	83	53	106	75	18	17
BACK PAIN ANY GRADE	49	21	NR	NR	14	14
INSOMNIA ANY GRADE	89	13	119	35	51	8
ABDOMINAL DISTENTION ANY GRADE	28	22	NR	NR	24	8
PALPITATIONS ANY GRADE	38	3	NR	NR	32	5
NASOPHARYNGITIS ANY GRADE	41	13	NR	NR	14	10
COUGH ANY GRADE	55	8	NR	NR	21	6
DIZZINESS ANY GRADE	61	13	NR	NR	24	11
HYPERTENSION ANY GRADE	71	8	NR	NR	20	1

NR, none reported;

† The category of fatigue includes reports of fatigue, asthenia, malaise and lethargy in all three studies;

* The category of thrombocytopenia includes reports of thrombocytopenia and decreased platelet count in all three studies.

Table S3. Grade 3 or 4 Side Effects of NOVA, PRIMA and NORA studies.

	**NOVA STUDY 2016**		**PRIMA STUDY 2019**		**NORA STUDY 2021**	
	Niraparib (n=367)	Placebo (n=179)	Niraparib (n=484)	Placebo (n=244)	Niraparib (n=177)	Placebo (n=88)
NAUSEA GRADE ¾	11	2	6	2	0	0
†FATIGUE GRADE ¾	30	1	9	1	1	0
ANEMIA GRADE ¾	93	0	150	4	26	2
*THROMBOCYTOPENIA GRADE ¾	124	1	202	2	20	1
VOMITING GRADE ¾	7	1	4	2	4	0
NEUTROPENIA GRADE ¾	72	3	62	3	36	7
HEADACHE GRADE ¾	1	0	2	0	1	0
DIARRHEA GRADE ¾	1	2	NR	NR	0	1
CONSTIPATION GRADE ¾	2	1	1	0	1	0
ABDOMINAL PAIN GRADE ¾	4	3	7	1	0	1
INSOMNIA GRADE ¾	1	0	4	1	1	0
ABDOMINAL DISTEN-TION GRADE ¾	0	1	NR	NR	1	0
HYPERTENSION GRADE ¾	30	4	NR	NR	2	0

NR, none reported;

† The category of fatigue includes reports of fatigue, asthenia, malaise and lethargy in all three studies;

* The category of thrombocytopenia includes reports of thrombocytopenia and decreased platelet count in all three studies.
